# *Metarhizium carneum* Formulations: A Promising New Biological Control to Be Incorporated in the Integrated Management of *Meloidogyne enterolobii* on Tomato Plants

**DOI:** 10.3390/plants12193431

**Published:** 2023-09-29

**Authors:** Daniel López-Lima, David Alarcón-Utrera, José Ángel Ordáz-Meléndez, Luc Villain, Gloria Carrión

**Affiliations:** 1 Facultad de Ciencias Agrícolas, Universidad Veracruzana, Xalapa 91000, Mexico; 2 Centre de Coopération Internationale en Recherche Agronomique pour le Développement (CIRAD), UMR DIADE, 34398 Montpellier, France; 3 Diversité, Adaptation et Développement des plantes (DIADE), Université de Montpellier, Pole Agriculture Environnement Biodiversité, IRD, CIRAD, 34394 Montpellier, France; 4 Instituto de Ecología, A.C. Red de Biodiversidad y Sistemática, Xalapa 91073, Mexico

**Keywords:** biological nematicide, plant-parasitic nematodes, integrated management, root-knot nematode

## Abstract

The increase in the populations of root-knot nematode *Meloidogyne enterolobii* in various vegetables such as tomatoes grown under greenhouse conditions as well as increasing restrictions on the use of certain chemical nematicides have led to the search for new, effective management strategies, preferably ones that are sustainable biological alternatives. In this work, two formulations of the nematophagous fungus *Metarhizium carneum*, one concentrated suspension and one wettable powder, were evaluated under greenhouse conditions to reduce the *M. enterolobii* infestation in tomato plants. In addition, the effectiveness of the liquid formulation of *M. carneum* was compared with two biological and three chemical commercial nematicides. The results show that the two *M. carneum* formulations reduced the *M. enterolobii* population density by 78 and 66% in relation to the control treatment. In comparison, the liquid formulation of *M. carneum* and *Purpureocillium lilacinum* treatments reduced nematode population density by 72 and 43%, respectively, while for metam sodium preplanting applications followed by *M. carneum* applications during the tomato growth stage, the reduction was 96%. The alternate use of some chemical compounds plus the application of *M. carneum* as a biocontrol is a good starting strategy for managing *M. enterolobii* populations. These results confirm that *M. carneum* is a serious candidate for the short-term commercialization of an environmentally friendly biological nematicide.

## 1. Introduction

Root-knot nematodes are the most widespread soilborne pathogens worldwide, causing USD millions in losses annually on various crops [1]. *Meloidogyne* species are sedentary endoparasites that induce feeding sites within the roots of their host, which allows the nematodes to grow and develop in interaction with the plant. The *Meloidogyne* life cycle begins with the second-stage juveniles (J2) hatching in the soil, which, upon finding a host plant, enter through the elongation zone of the roots and migrate towards the vascular cylinder [2], where they induce the differentiation of some cells into multinucleated cells by hyperplasia. These giant cells are metabolically very active and serve as a constant source of food for the nematode. Giant cells at feeding sites cause root gall symptoms [3]. *Meloidogyne enterolobii* is considered an emerging nematode species of major concern due to its worldwide distribution and its ability to reproduce on various crops, causing severe damage [4]. This nematode was recorded for the first time in Mexico parasitizing watermelon plants in the state of Veracruz [5], and later it was registered in tomato cultivated fields in Sinaloa, the main vegetable-producing state, causing severe damage to tomato plants, even those carrying the Mi gene which provides resistance to *M. incognita*, *M. javanica* and *M. arenaria* [6,7]. This is of particular concern because Mexico is the tenth largest tomato producer at four million tons per year, and it is also the main exporter worldwide [8,9]. Currently, *M. enterolobii* is reported in Mexico on cucumber [10], pepper [11], eggplant [12], carrot and beet crops [13]. The ability of *M. enterolobii* to infect various plant species and overcome host resistance mechanisms is one of the main problems for field management [14,15]. The control of the *M. enterolobii* population is carried out using chemical nematicides, mainly organophosphates and carbamates. However, these nematicides have drawbacks since they must be applied in high doses and repeatedly to keep the nematode population below the economic threshold [16]. In addition, the constant use of these chemical nematicides leads to their ineffectiveness after prolonged use and causes harmful effects on beneficial organisms along with contamination of groundwater; therefore, restrictions were implemented for their use in the control of plant-parasitic nematodes [16,17].

The harmful effects of chemical nematicides can be overcome by using biological control, which is a safer, more effective and environmentally friendly alternative. Some nematophagous fungi have been used as biological control agents since they can modify their saprophytic behavior to feed on nematodes, developing various strategies to infect them [18]. The nematophagous fungus *Metarhizium carneum* (=*Paecilomyces carneus*) (Duché & R. Heim) Kepler, S.A. Rehner & Humber) was recorded as a parasite of *Meloidogyne* spp. [19,20], *Heterodera avenae* [21,22] and *Globodera rostochiensis* [20]. The biological effectiveness of *M. carneum* against nematodes was evaluated with the application of concentrated conidia in suspension [20]; however, its performance when manufactured as a commercial product is unknown. Likewise, it is important to compare its effectiveness with respect to other nematicides, both biological and chemical, available on the market. In considering the potential of using *M. carneum* as a nematicide, the aims of this work are as follows: (1) to evaluate the effects of two formulations of *M. carneum* in liquid and powder forms in tomato plants infected with *M. enterolobii*, and (2) to compare their effectiveness with other commercial nematicides.

## 2. Results

### 2.1. Evaluation of the Liquid vs. Powdered Formulation of Metarhizium carneum against Meloidogyne enterolobii in Greenhouse Tomato Plants

The tomato plants treated separately with one of the two formulations of *M. carneum* presented similar numbers of *M. enterolobii* galls: an average of 145 and 156 galls for the liquid and powder formulations, respectively. These values were not significantly different from the 189 average galls observed in the untreated plants. Although the galling index was similar in the three treatments, the galls registered in the plants treated with the liquid formulation of *M. carneum* presented a smaller size. Moreover, the two formulations, liquid and powder, reduced significantly (*p* ≤ 0.01) the population of *M. enterolobii* (eggs and J2) in the roots of tomato plants by 78 and 66%, respectively, with respect to the control (Table 1).

Population density of J2 in the rhizospheric soil was very low. The total populations of nematodes found in *M. carneum* treatments were significantly lower than the population found in the control (Table 1). Based on the final population of nematodes in the roots and in the rhizospheric soil, the reproduction factor in the liquid and powder treatments was 4.8 and 4.2, respectively, in contrast to the reproduction factor of 14.6 in the untreated plants. About the plants’ development, similar values were found in the three treatments. However, the tomato plants treated with the liquid formulation of *M. carneum* showed a greater foliage fresh weight (Table 2).

### 2.2. Effectiveness of M. carneum Liquid Formulation Compared to Commercial Nematicide Products

The evaluations carried out at 60 and 120 days after tomato seedling transplanting allowed us to observe how the population of nematodes was reduced or increased according to the treatment. At 60 days after transplanting, the plants treated with metam sodium + *M. carneum*, fluensulfone and metam sodium + abamectin did not show galling symptoms, while the plants treated separately with *M. carneum*, *Purpureocillium lilacinum* and the inactivated ferment of *Myrothecium verrucaria* presented slight galling with no necrosis signs. Control group plants showed symptoms of medium galling with the beginning of necrotic areas. The population density of *M. enterolobii* in the roots of the plants treated with metam sodium + *M. carneum*, fluensulfone and metam sodium + abamectin were significantly (*p* ≤ 0.01) lower than the rest of the treatments, with a reduction of 99, 98 and 99%, respectively, compared to the control treatment; therefore, the reproduction factor was also significantly lower (Table 3). Likewise, in the treatments with *M. carneum*, *P. lilacinum* and the inactivated *M. verrucaria* ferment, the nematode population density was significantly (*p* ≤ 0.01) lower than population density in the control treatment, with a reduction of 28, 39 and 61%, respectively. The nematode reproduction factor in the last three treatments was of 6.2, 6.6 and 4.6, respectively, significantly lower than the reproduction factor in the control treatment, which was 9.8 (Table 3). In addition, the plants treated with the inactivated *M. verrucaria* ferment had greater (*p* ≤ 0.01) foliage fresh weight, flower number and chlorophyll content than untreated plants (Table S1).

**Figure 1:**
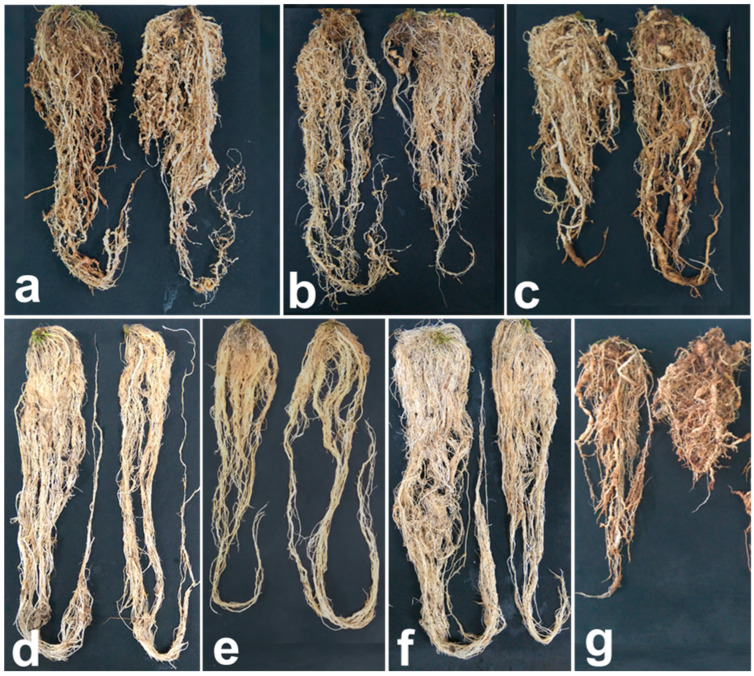
Tomato roots infested with *Meloidogyne enterolobii* at 120 days after transplanting under different nematicide treatments: (**a**) liquid formulation of *Metarhizium carneum*, (**b**) *Purpureocillium lilacinum*, (**c**) metabolites from the fermentation of *Myrothecium verrucaria*, (**d**) metam sodium + *Metarhizium carneum*, (**e**) fluensulfone, (**f**) metam sodium + abamectin and (**g**) untreated control.

At 120 days after transplanting, the roots of the plants treated with *M. carneum* showed a medium to high galling index, with continuous thickening and the beginnings of necrosis; however, in this treatment, the root system was very abundant despite heavy infestation. Regarding the plants treated with *P. lilacinum* and the inactive ferment of *M. verrucaria*, the galling index was strong to severe, with continuous thickening, cracking in the root cortex necrosis and a reduced root system. The roots of the plants treated with fluensulfone presented slight necrosis of the older roots, and the root system was slightly reduced in weight, while the plants treated with metam sodium + *M. carneum* and metam sodium + abamectin presented a galling index of zero to light (Figure 1).

**Figure 2:**
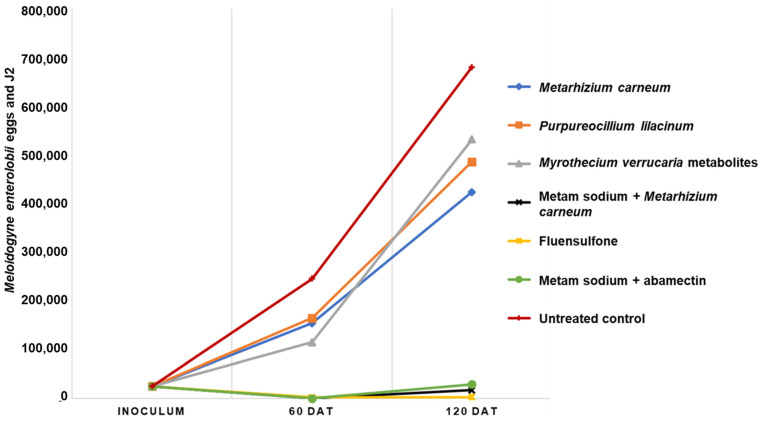
Evolution of *Meloidogyne enterolobii* population on tomato plants under different nematicide treatments. DAT = days after transplant.

**Figure 3:**
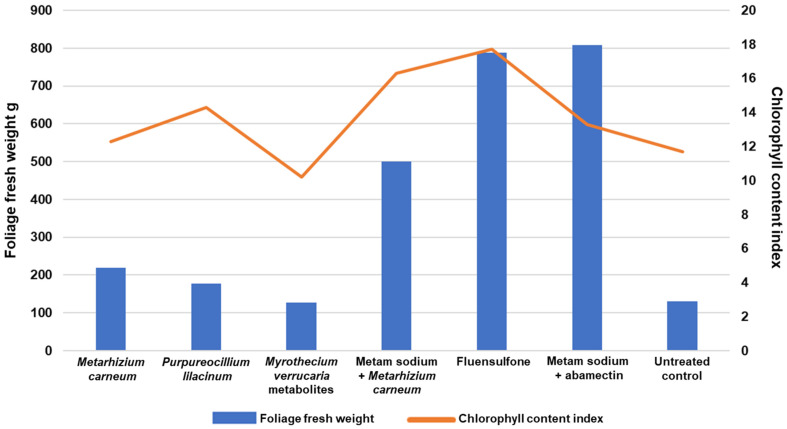
Foliage fresh weight and chlorophyll content at 120 days after transplanting of tomato plants infested with *Meloidogyne enterolobii* and under different nematicide treatments.

Plants treated with *M. carneum* had the lowest (*p* ≤ 0.01) root population density (72%) compared with biological treatments and control plants, followed by plants treated with *P. lilacinum* with a reduction of 43%. On the other hand, plants treated with the metabolites of *M. verrucaria* fermentation presented a high final population density, only 2% less than the untreated plants. The lowest population density of *M. enterolobii* was recorded in the roots of the plants treated with metam sodium + *M. carneum*, fluensulfone and metam sodium + abamectin with a reduction compared with the control group of 96, 99 and 97%, respectively. The reproduction factor of *M. enterolobii* in treatments with *M. carneum*, *P. lilacium* and the inactive ferment of *M. verrucaria* reached values of 17, 19.5 and 21.3, respectively, which are not significantly different (*p* ≤ 0.01) from the value of 27.2 reached for the control group (Table 4). Likewise, the reproduction factor and final population was significantly lower in the metam sodium + *M. carneum*, fluensulfone and metam sodium + abamectin treatments (Table 4, Figure 2). Likewise, the plants treated with these three treatments developed better in terms of higher biomass, chlorophyll content (Figure 3), number of flowers and root length (Table S2).

## 3. Discussion

Currently, *M. enterolobii* is considered an important risk for agricultural production due to its worldwide distribution and wide host range. Moreover, this species is recognized as one of the most virulent root-knot nematodes because of its ability to reproduce in plants with resistance to the main root-knot nematode tropical species [4,23]. Several methods, such as chemical nematicides, biological control, resistant cultivars and cultural control methods, have recently been evaluated in the control of *M. enterolobii* [24]. The results of this work contribute to the design of integrated pest management strategies that include the application of various control methods to increase efficiency in damage reduction and reduce the pathogen population below the economic threshold [25].

In Mexico, this nematode causes significant yield losses in tomato and other vegetables crops due to its high pathogenicity, which leads to important plant mortality before completing the crop cycle. Even tomato cultivars with the Mi-1 resistance gene, which confers resistance to *M. javanica*, *M. arenaria* and *M. incognita*, are susceptible to *M. enterolobii* [6,7]. Therefore, in this work, the research focused on the development of integrated management strategies for this emerging species using biological and chemical controls.

The effectiveness of the application of bioproducts of nematophagous fungi was previously demonstrated in other *Meloidogyne* species and in other crops [26,27,28], with *Purpureocillium lilacinum* being the most widely used nematophagous fungus worldwide, and there are currently many products available on the market developed with its conidia [29,30,31,32,33]. However, in recent years, there have been cases of severe infections in humans, so it is necessary to evaluate the nematicidal properties of other species of fungi to provide other, safer biological control alternatives for farmers’ health [34,35].

Fungi of the *Metarhizium* genus, commonly used for their entomopathogenic activity, were shown to be effective in parasitizing the root-knot nematode *M. incognita* [36]. In particular, *M. carneum* showed its effectiveness in reducing populations of the potato cyst nematode *G. rostochiensis* by 33 to 89% under field conditions [20]. The results of this work confirm the effectiveness of *M. carneum* formulations obtained by reproducing it in a bioreactor (active fermentation) or on a solid substrate for the reduction of *M. enterolobii* populations. The way the microorganisms used as biological control agents are reproduced is an important factor in their performance in crop fields. Various studies indicate that liquid formulations may have advantages over powder ones because the spores remain viable for more time. There is also less risk of contamination compared to powder presentations, and therefore the liquid presents greater efficiency in reducing populations of plant-parasitic nematodes [37,38,39]. Moreover, it was indicated that microorganisms reproduced in liquid media have greater efficiency potential since the microorganisms remain in a latent stage and become active only when applied to the soil [40]. The results of this work reveal that the two ways of reproducing *M. carneum* have a similar potential in the suppression of *M. enterolobii*. However, the liquid formulation provides a higher concentration of spores in less time, and therefore, this means that smaller quantities of product can be applied per hectare.

The early application of *M. carneum* in the field, before transplanting or planting, is an important factor to consider in the integrated management of pathogenic nematodes, as has been carried out in fields heavily infested with *G. rostochiensis* [20]. This way, the fungus has more opportunity to parasitize eggs and J2. Conversely, when the nematicide was applied seven days after nematode inoculation, many J2 were allowed to penetrate roots and establish their feeding site. The process of root infestation and feeding-site establishment by the nematode takes approximately 10 days [3]. For this reason, the number of galls and the galling index in the plants with the application of the two *M. carneum* formulations (powder and liquid) were like those of the inoculated control, although nematode root population density was very different. This also indicates that the evaluation of the number of galls or the galling index are not adequate references when evaluating the nematicidal properties of any product. Based on our data, we think that to efficiently control the population density of *M. enterolobii* in tomato roots in soils highly infested by nematodes, it is important to reduce the population before transplanting, when nematodes are still at egg and J2 stages. *Metarhizium carneum* was recorded as a parasite of nematode immobile stages such as eggs and females of *Globodera*, *Heterodera* and *Meloidogyne* [19,21,22], because of its ability to cross the nematode’s cuticle and develop its mycelium inside [20]. In this work, both formulations (liquid and powder) significantly reduced the population in the soil. In the genus *Metarhizium*, other species were recorded to produce spores with a thin mucilaginous layer that allowed them to adhere to the surface of their host to form an appressorium, from which a narrow hypha was produced that penetrated the cuticle using a combination of enzymes and mechanical strength [41]. It is possible that *M. carneum* has a similar adhesion mechanism, which would explain its suppressive effect on J2 in the soil; however, further research is needed.

In the second trial comparing the effectiveness between the *M. carneum* liquid formulation and commercial biological and chemical nematicides, both treatments with the application of *M. carneum* and *P. lilacinum* allowed a reduction in the nematode population. Novel biorational strategies have been evaluated for the *M. enterolobii* control. For example, *P. lilacinum* and *Pochonia chlamydosporia* conidia reduced by 80% the hatching of this nematode under in vitro conditions [14]; *Aspergillus tubingensis* fermentation inhibited *M. enterolobii* hatching and caused 100% mortality of its J2 in in vitro conditions and significantly reduced the infestation of greenhouse tomato plants [42]; and indole 3-butyric acid prevented *M. enterolobii* infestation in guava plants [43]. Although the applications of *M. carneum* reduce the population of *M. enterolobii* in heavily infested soils, it may be interesting to use this biological control with other novel control strategies such as those described above and also including crop rotation and the use of resistant cultivars to reduce the nematode population in less time [20]. Furthermore, it is important to consider that in addition to the nematicidal properties of biological control agents, their effectiveness also depends on other factors such as the formulation viability, crop management and environmental conditions at the application time [44].

The application of the inactivated metabolites of *M. verrucaria* fermentation showed better performance at 60 days after transplanting. In other studies, it was found to inhibit eggs from hatching by a nematostatic effect on the J2 of *M. javanica*, preventing early root invasion [45,46]. Application of this nematicide also reduced galling in beet plants infested by *M. incognita* by up to 86% [47]. However, in the 120 days after transplanting evaluation of our study, it performed poorly and matched the untreated control population. The low effect of the *M. verrucaria* product 120 days after transplanting may be due to the severe thickening of the roots, which causes the juveniles to not leave the root tissue towards the ground but instead to reinfest the plant root in the same area, as was observed in severe infestations of *Meloidogyne paranaensis* in coffee roots [48]. So, the J2 juveniles are not exposed to the product that has a nematostatic effect, which therefore does not carry out the control; however, more studies are needed to confirm this hypothesis. Based on the results of this work, the metabolites from the fermentation of *M. verrrucaria* could be used in alternating applications with a nematophagous fungus to affect all stages of the nematode, but it is necessary to carry out the corresponding evaluations.

One of the practices for controlling nematodes in conventional agriculture is soil disinfection with chemicals to reduce the nematodes’ number as much as possible before transplanting. In this work, this strategy was used, applying two chemical compounds in the pretransplanting phase (fluensulfone and metam sodium). Various studies indicate that the most efficient nematicides in reducing the populations of various *Meloidogyne* species are soil fumigants, such a 1,3 dichloropropene, chloropicrin and metam sodium, since they can have an effectiveness between 50 and 80% in decreasing the initial nematode populations in the soil [49,50,51]. This coincides with what was recorded in this work, where the application of metam sodium, a chemical from the ditiocarbamate group, resulted in very low infestations in the tomato roots. However, it is known that the application of this chemical nematicide to the soil also reduces the abundance and diversity of communities of beneficial bacteria, fungi and non-plant-parasitic nematodes, among others, so the continuous use of this chemical nematicide over crop cycles significantly affects the microbial balance of the rhizosphere [52,53]. In addition to this, the most common practice in conventional agriculture is to continue nematode control with post-transplant applications of chemical nematicides after initial preplanting soil fumigation, which increases the damage to edaphic biota. In this work, it was shown that fumigation with metam sodium before transplanting and then the use of *M. carneum* during the growth stage of the crop has the same effectiveness as using a post-transplant chemical nematicide, so this procedure is a good alternative that can reduce the application of chemical nematicides in highly infested soils.

The nonfumigant nematicide applied to pretransplant fluensulfone is one of the active ingredients with the greatest potential to reduce nematode populations. Its effectiveness has been verified against *M. enterolobii*, where it significantly reduces damage to sweet potato and tobacco [54,55]. Although it is classified as slightly toxic to humans and various studies indicate low toxicity towards nontarget organisms [16], the impact of constant applications should be studied. Fluensulfone has also been found to have phytotoxic effects on many cultivated plants [56]. In particular, the sensitivity of tomato plants to this nematicide has been pointed out; however, in this work, no significant delay in the development of plants treated with fluensulfone was observed. Chemical nematicides used in conventional agriculture are important since they can significantly reduce the nematode populations, allowing crops to maintain their productivity; however, due to the registered consequences of continuous use, it is necessary to have environmentally friendly alternatives such as bionematicides.

## 4. Materials and Methods

### 4.1. Fungal Strain and Growth Conditions

The fungal used in this study was *M. carneum* strain IE-431 stored at the Instituto de Ecología A.C. (INECOL) strain collection, registered with the World Federation for Culture Collection (No. IEWDCM 782) and the Chilean Collection of Microbial Genetic Resources (CChRGM). The IE-431 strain was grown on potato dextrose agar (PDA) medium plates and incubated at 22 °C for 10 days. Subsequently, a collection of spores was made using 50 mL of sterile distilled water with Tween 80 at 0.01%, adjusting the spore concentration by using a Newbauer chamber for use in preparing formulations.

### 4.2. Preparation of Metarhizium carneum Formulations

To prepare the *M. carneum* formulation, a preinoculum of the fungus was put into a flask of 1 L with 500 mL of potato dextrose broth (PDB) at a concentration 1 × 10^6^ conidia mL^−1^. The flask was incubated at 22 °C and at 100 rpm for 72 h. For the liquid formulation, the fungus was reproduced in a BioFlo 415 bioreactor (New Brunswick Scientific, Edison, NJ, USA) with a capacity of 10.5 L (Eppendorf™, Hamburg, Germany) with an oat broth as the growth medium [20]; 50 mL of the preinoculum was added to the bioreactor and was incubated for 120 h. Finally, a stabilizer was added to the ferment, which was kept in a hermetically sealed 1 L flask at room temperature. For the powdered formulation, 200 g of sterilized semistarchy rice was impregnated with 25 mL of preinoculum of *M. carneum* inside a high-density polyethylene bag and was then incubated for 30 days at room temperature (22 ± 2 °C and 60% relative humidity). Afterwards, the rice with the fungus developed was shredded with a food processor (Ninja Intelli-Sense CT680CO2SS, Ninja Kitchen, Newton, MA, USA) at top speed for 20 s. The obtained powder was mixed with 20% of diatom (Dia-Fix™ Zeolitech, Cuernavaca, Mexico). The mixture was then stored in vacuum-sealed bags and kept at room temperature (22 ± 2 °C). At the time of the sealing process, the fermentation suspension had 1.8 × 10^8^ CFU mL^−1^ and the powder had 1.8 × 10^7^ CFU g^−1^.

### 4.3. Obtaining Meloidogyne enterolobii Inoculum

The eggs and juveniles J2 of *M. enterolobii* came from a population collected in tomato plants in the Sinaloa state, Mexico, which were previously identified molecularly by species-specific SCAR markers and which multiplied in tomato plants under greenhouse conditions [57].

### 4.4. Evaluation of Metarhizium carneum Formulations for Controlling Meloidogyne enterolobii in Tomato

Tomato plants cv. Rio Grande with a pair of leaves after the cotyledon 20 days after germination (obtained from seed germination in sterile substrate) were transplanted into polypropylene pots (20 cm in diameter) with 5 L of substrate composed of soil, sand, vermiculite and peatmoss (20:15:15:50). The substrate was previously sterilized in a soil pasteurizer with 380 L in capacity (Pro-Grow™ SS-60). Five days after transplanting, each plant was inoculated with 17,500 eggs and J2 of *M. enterolobii* contained in a suspension of 50 mL of water that was applied directly in three holes at the stem base with a 10 mL automatic micropipette (Eppendorf™ I18057D) to achieve an inoculation of 3.5 nematodes per cm^3^ of substrate to ensure the nematode establishment. To evaluate the bioformulates, the following treatments were performed: (1) liquid formulation of *M. carneum*, (2) powder formulation of *M. carneum* and (3) control group also inoculated with *M. enterolobii*, without application of *M. carneum* or any nematicidal treatment. Both presentations of *M. carneum* were applied in a dose equivalent to 1.8 × 10^11^ CFU ha^−1^ seven days after the nematode inoculation, then six times at 15-day intervals. Each treatment was made up of 15 plants, and each plant was considered as a repetition in a completely randomized design. Tomato plants were removed from the pots 120 days after nematode inoculation. The rhizospheric soil was removed with a fine-bristled brush and preserved. The roots were gently washed under running water and placed on absorbent paper to remove excess water and evaluate their length and fresh weight. Plant heights and foliage weights were also evaluated. For each root, gall number was assessed, and the galling index was determined according to the Coyne and Ross scale [58]. For eggs and J2 extraction from plant tissue, the entire root system was cut into 1–2 cm fragments and processed using the crushing, sieving and centrifugation technique [59]. Further, nematodes were also extracted from the 100 g of rhizospheric soil using the sieving–centrifugation technique [60]. Nematodes extracted from plant roots and soil were both quantified in a Sedgwick–Rafter chamber under a 100× light microscope. The population density of nematodes in roots and rhizospheric soil, the total population in roots and soil as well as the reproduction factor (final population/initial population) were calculated.

### 4.5. Evaluation of Metarhizium carneum Liquid Formulation Compared to Different Commercial Nematicide Products for Controlling Meloidogyne enterolobii in Tomato

The pot substrate was prepared in the same way as in the previous experiment. The pots were inoculated with 25,000 viable *M. enterolobii* eggs to obtain an infestation level of 5 eggs per cm^3^ soil^−1^, consistent with the high infestation levels observed in horticultural soils in Mexico [10,12,61]. Ball tomato plants cv. Horus with a pair of leaves after cotyledons formation, also obtained from seed germination in sterile substrate (20 days after germination), were transplanted to the pots 32 days after the nematode inoculation. The experiment consisted of seven treatments, as follows, with 18 repetitions (one plant per pot) distributed in a completely randomized design: (1) Liquid formulation of *M. carneum* (1 × 10^8^ CFU mL^−1^) developed in this work at a dose of 2 L ha^−1^. (2) Commercial product of nematophagous fungus *P. lilacinum* (Lila-Plus™ Arysta, 1.2 × 10^12^ conidia 240 g^−1^, wettable powder) at a dose of 480 g ha^−1^; treatments (1) and (2) were applied 30 days before transplanting and at planting time and then had six subsequent applications at 15-day intervals. (3) Inactive fermentation of the fungus *Myrothecium verrucaria* with nematostatic activity (DiTera DF™, Valent 90% a.i.) at a dose of 2.5 Kg ha^−1^ starting at the planting and every 10 days on nine occasions. (4) Metam sodium (BL 1480™, Buckman Laboratories 42% a.i.) at a dose of 400 L ha^−1^ 30 days before the transplant and continuing with applications of liquid formulation of *M. carneum* (1 × 10^8^ CFU mL^−1^) at a dose of 2 L ha^−1^ starting at the planting time and om six subsequent applications at intervals of 15 days. (5) Fluensulfone chemical nematicide (5-Chloro-2-((3,4,4-trifluorobut-3-en-1-yl) sulfonyl) thiazole; Nimitz™ 480 EC Adama, 480 g a.i. L^−1^) at a dose of 2 L ha^−1^ 30 days before transplanting for a single occasion. (6) Metam sodium (BL 1480™, Buckman Laboratories 42% a.i.) at a dose of 400 L ha^−1^ 30 days before transplanting and continuing with abamectin (Oregon™ 60 SC FMC, 60 g a.i. L^−1^) at a dose of 1.25 L ha^−1^ on six occasions, starting at the planting, at intervals of 15 days. Metam sodium and abamectin are commonly used by farmers om the management of *Meloidogyne* populations. Therefore, we consider this treatment as conventional management. (7) Untreated control, without nematicides application. All products were applied in accordance with the manufacturer’s recommendations. The application of the pretransplant products was carried out 48 h after nematode inoculation. Two evaluations were carried out, one at 60 and another at 120 days after transplanting, using nine repetitions (plants) by treatment at each evaluation time. The galling index was measured according to the Coyne and Ross scale [58], and the number of nematodes per root system was quantified to obtain the nematode root population density, the final root population and the reproduction factor using the same methodology as in the first experiment (Section 4.4). In this experiment, unlike the first experiment, the amount of J2 in the rhizospheric soil was not evaluated because it is a residual population and does not provide valuable information, as can be seen in Table 1. Also, the foliage fresh weight (g), number of flowers and chlorophyll content were measured, the latter using the portable chlorophyll meter CL-01 (Hansatech™ Instruments Ltd., Norfolk, UK), which determines the relative chlorophyll content of the leaves by measuring the absorbance at the wavelengths (660 and 940 nm); the values are expressed in CCI (chlorophyll content index) units.

### 4.6. Greenhouse Conditions

All of the experiments were conducted in the greenhouse in the Planta Piloto de Desarrollo de Agentes de Control Biológico at the Instituto de Ecología A.C. under natural light conditions at a temperature of 25 ± 3 °C. The plants were under a drip irrigation system (up to 1.2 L per day). In addition, 10 g of 18-46-0 granular fertilizer (Fertigolfo™, Banderilla, Mexico) was added to each plant plus 50 mL 0.14% 20-20-20 water-soluble fertilizer (Peters™ Israel Chemicals Ltd., Tel Aviv-Yafo, Israel) applied three times. During plant growth, to prevent fungal diseases, copper hydroxide (Cupravit Hidro™ Bayer, Barmen, Germany) and metalaxyl + chlorothalonil (Fungoxil™ United Phosphorus de Mexico) were applied to the foliage at 30 and 75 days after transplanting. An antibacterial pentahydrated copper sulfate + gentamycin sulfate + oxytetracycline chloride (Genoxi™ Lapisa, La Piedad, Mexico) was also added 5 times, every 15 days starting from day 15. The insecticide flupyradifurone (Sivanto™ Bayer, Barmen, Germany) was applied during the 45th and 75th day after transplant to suppress white flies.

### 4.7. Statistical Analysis

Nematode count data were transformed (log 10) before analysis [62]. All the data were analyzed using a one-way analysis of variance (ANOVA) and a Tukey test for treatment ranking. Significant differences were considered at *p* ≤ 0.01. All analyses were performed with the Statistica 12.5 package for Windows.

## 5. Conclusions

Based on the results of this work, we can conclude that liquid and powder formulations made with *M. carneum* are effective for controlling *M. enterolobii* populations in tomato plants. Thus, a root-knot nematode control strategy that starts with preplanting soil disinfection using chemical nematicides can be complemented by effective biological control during the development of the tomato crop through applications of *M. carneum* until shortly before harvest without causing health issues due to residues in the harvested tomatoes. Our results support the purpose of successfully incorporating biological products into nematode management packages to transition to sustainable agriculture.

## Figures and Tables

**Table 1:** Population of *Meloidogyne enterolobii* (mean ± standard deviation) in tomato plants treated with liquid and powder formulations of *Metarhizium carneum*.

Treatments	Galls Number per Root	Galling Index	Eggs and J2 g Root^−1^	J2 g^−1^ Rhizospheric Soil	Total Nematode Population
Liquid	145 ± 110	1.86 ± 0.4	14,481 ± 11,938 a ^1^	12 ± 17 a	85,005 ± 75,363 a
Powder	156 ± 124	1.93 ± 0.26	22,207 ± 13,505 a	11 ± 12 a	74,678 ± 57,624 a
Untreated	189 ± 106	2 ± 0	64,791 ± 38,944 b	26 ± 8 b	257,187 ± 170,414 b
*p*	*p* = 0.66	*p* = 0.72	*p* < 0.01	*p* < 0.01	*p* < 0.01

^1^ Different letters in each column denote significant differences between treatments indicated by a Tukey test; *p* ≤ 0.01.

**Table 2:** Development of tomato plants infested with *Meloidogyne enterolobii* treated with liquid and powder formulations of *Metarhizium carneum* (mean ± standard deviation).

Treatments	Height cm	Foliage Fresh Weight g	Rooth Length cm	Root Fresh Weight g
Liquid	97 ± 10	46 ± 10 a ^1^	19.5 ± 4.1	5.2 ± 3.6
Powder	89 ± 15	36 ± 10.9 b	16.4 ± 4.3	3.7 ± 1.9
Control	90 ± 13	40 ± 10 ab	16.8 ± 3.8	4.6 ± 2.6
*p*	0.21	0.01	0.08	0.09

^1^ Different letters in each column denote significant differences between treatments indicated by a Tukey test; *p* ≤ 0.01.

**Table 3:** *Meloidogyne enterolobii* symptoms and populations (mean ± standard deviation) at 60 days after transplanting on tomato plants under different nematicide treatments.

Treatments	Galling Index	Eggs and J2 g Root^−1^	Final Eggs and J2	Reproduction Factor
*Metarhizium carneum*	2.6 ± 0.5 b ^1^	6665 ± 2453 cd	154,542 ± 37,516 b	6.2 ± 1.5 b
*Purpureocillium lilacinum*	2.6 ± 0.5 b	5626 ± 1045 bc	164,850 ± 28,727 b	6.6 ± 1.1 b
Metabolites from *M. verrucaria* fermentation	2.3 ± 0.5 b	3553 ± 2020 b	115,238 ± 58,897 b	4.6 ± 2.3 b
Metam sodium + *Metarhizium carneum*	1.0 ± 0 a	66 ± 19 a	563 ± 194 a	0.02 ± 0.007 a
Fluensulfone	1.0 ± 0 a	158 ± 59 a	1913 ± 1017 a	0.1 ± 0.04 a
Metam sodium + abamectina	1.0 ± 0 a	15 ± 4 a	128 ± 22.5 a	0.005 ± 0.0009 a
Control	3.3 ± 0.5 c	9255 ± 2355 d	245,625 ± 26,591 c	9.8 ± 1 c
*p*	<0.01	<0.01	<0.01	<0.01

^1^ Different letters in each column denote significant differences between treatments indicated by a Tukey test; *p* ≤ 0.01.

**Table 4:** *Meloidogyne enterolobii* symptoms and population (mean ± standard deviation) at 120 days after transplanting on tomato plants under different nematicide treatments.

Treatments	Galling Index	Eggs and J2 g Root^−1^	Final Eggs and J2	Reproduction Factor
*Metarhizium carneum*	3.4 ± 0.5 b ^1^	2614 ± 1298 ab	423,825 ± 168,141 b	17.0 ± 6.7 b
*Purpureocillium lilacinum*	4.3 ± 0.5 c	5296 ± 2344 b	486,300 ± 133,878 b	19.5 ± 5.4 b
Metabolites from *M. verrucaria* fermentation	4.7 ± 0.4 c	9049 ± 3733 c	532,425 ± 127,316 b	21.3 ± 5.1 b
Metam sodium + *Metarhizium carneum*	1.3 ± 0.5 a	336 ± 12.2 a	16,980 ± 5087 a	0.7 ± 0.2 a
Fluensulfone	1.0 ± 0 a	83 ± 33 a	2625 ± 1267 a	0.1 ± 0.05 a
Metam sodium + abamectina	1.3 ± 0.5 a	312 ± 212 a	29,175 ± 12,862 a	1.2 ± 0.5 a
Control	5.0 ± 0 c	9268 ± 2111 c	681,000 ± 531,754 b	27.2 ± 21.3 b
*p*	<0.01	<0.01	<0.01	<0.01

^1^ Different letters in each column denote significant differences between treatments indicated by a Tukey test; *p* ≤ 0.01.

## Data Availability

Data are available upon request to the corresponding author.

## Supplementary Materials

The following supporting information can be downloaded at: https://www.mdpi.com/article/10.3390/plants12193431/s1, Table S1: Development (mean ± standard deviation) at 60 days after transplanting of tomato plants infested with *Meloidogyne enterolobii* and under different nematicide treatments; Table S2: Development (mean ± standard deviation) at 120 days after transplanting of tomato plants infested with *Meloidogyne enterolobii* and under different nematicide treatments.
