# Supplementary material for: Metarhizium carneum Formulations: A Promising New Biological Control to Be Incorporated in the Integrated Management of Meloidogyne enterolobii on Tomato Plants
Source: Plants (Basel). 2023 Sep 29;12(19):3431. doi: 10.3390/plants12193431 (PMC10574380; doi:10.3390/plants12193431)
Supplement: Supplementary file 1 [file plants-12-03431-s001.zip › plants-2592741-supplementary.pdf]

Table S1. Development (mean ± standard deviation) at 60 days after transplanting of tomato plants infested with *Meloidogyne enterolobii* and under different nematicide treatments.

**Treatments**	**Height cm**	**Stem diameter cm**	**Foliage fresh weight g**	**Flowers number**	**Chlorophyll content index**	**Root length cm**	**Root weight g**
Metarhizium carneum	92±3 ab	7.0±0.9 a	86.6±5.7 bc	10.3±3.9 b	18.7±2.5 b	55.4±5.5	29.2±1 a
Purpureocillium lilacinum	82.7±11 b	6.5±0,2 abc	72.7±10 c	5.7±3 b	17.4±2 b	44.0±11	29.4±0.4 a
Metabolites from *M. verrucaria* fermentation	106.7±6.7 a	6.7±0.5 ab	114.9±4.2 a	20.7±4.4 a	28.9±6.6 a	63.3±16	33.4±3 a
Metam sodium + *Metarhizium carneum*	79.3±6.5 b	5.2± 0.4d	75.5±5.8 c	5.0±0.8 b	21.8±3.7 b	48.7±6	8.5±0.7 b
Fluensulfone	97.0±22 ab	5.7±0.5 cd	100.2±26 ab	10.3±6.1 b	18.7±1.1 b	56.2±35	11.1±3.1 b
Metam sodium + abamectina	89.7±2.7 ab	5.4±0.5 d	82.3±8.1 bc	4.7±1.3 b	18.9±3.2 b	55.3±13	8.9±2.1 b
Control	97.3±17 ab	5.8±0.6 bcd	82.6±20 bc	9.0±4.5 b	18.4±4.2 b	53.5±9.9	28.3±8.1 a
p	<0.01	<0.01	<0.01	<0.01	<0.01	0.34	<0.01

Different letters in each column denote significant differences between treatments indicated by a Tukey test p≤ 0.01.

Table S2. Development (mean ± standard deviation) at 120 days after transplanting of tomato plants infested with *Meloidogyne enterolobii* and under different nematicide treatments.

**Treatments**	**Height cm**	**Stem diameter cm**	**Foliage fresh weight g**	**Flowers number**	**Chlorophyll content index**	**Root length cm**	**Root weight g**
Metarhizium carneum	161.8±18.1 bc	9.3±0.2 ab	218.6±17 c	14.7±1.8 b	12.3±3.5 bc	55.1±19 bc	169.6±28 a
Purpureocillium lilacinum	164.7±35 bc	8.5±1.5 ab	177.2±95 c	8.7±3.5 b	14.3±2.5 abc	43.8±19 c	105.7±36 ab
Metabolites from *M. verrucaria* fermentation	115.7±24 d	8.4±1.1 ab	126.9±84 c	12.3±7 b	10.2±3.3 c	38.7±14 c	71.2±35 bc
Metam sodium + *Metarhizium carneum*	159.0±27 cd	7.8±0.1 b	500.6±156 b	16.3±8.6 b	16.3±2.5 ab	82.0±12 a	50.6±15 bc
Fluensulfone	206.0±33 ab	8.6±0.5 ab	788.4±40 a	41.7±20 a	17.7±2.8 a	71.0±13 b	31.0±5.9 c
Metam sodium + abamectina	208.5±29 a	9.5±0.8 a	808.0±173 a	53.3±14.5 a	13.3±0.8 abc	78.4±13 ab	107.1±33 ab
Control	149.5±13 cd	8.5±1.3 ab	130.9±72 c	9.3±8.7 b	11.7±3.8 bc	43.6±2.7 c	90.9±89.1 bc
p	<0.01	<0.01	<0.01	<0.01	<0.01	<0.01	<0.01

Different letters in each column denote significant differences between treatments indicated by a Tukey test p≤ 0.01.
